# Necroptosis protects against exacerbation of acute pancreatitis

**DOI:** 10.1038/s41419-021-03847-w

**Published:** 2021-06-10

**Authors:** Michittra Boonchan, Hideki Arimochi, Kunihiro Otsuka, Tomoko Kobayashi, Hisanori Uehara, Thiranut Jaroonwitchawan, Yuki Sasaki, Shin-ichi Tsukumo, Koji Yasutomo

**Affiliations:** 1 Department of Immunology and Parasitology, Graduate School of Medicine, Tokushima University, Tokushima, Japan; 2 Department of Interdisciplinary Researches for Medicine and Photonics, Institute of Post-LED Photonics, Tokushima University, Tokushima, Japan; 3 Division of Pathology, Tokushima University Hospital, Tokushima, Japan; 4 Research Cluster Program on Immunological Diseases, Tokushima University, Tokushima, Japan

**Keywords:** Acute pancreatitis, Experimental models of disease

## Abstract

The sensing of various extrinsic stimuli triggers the receptor-interacting protein kinase-3 (RIPK3)-mediated signaling pathway, which leads to mixed-lineage kinase-like (MLKL) phosphorylation followed by necroptosis. Although necroptosis is a form of cell death and is involved in inflammatory conditions, the roles of necroptosis in acute pancreatitis (AP) remain unclear. In the current study, we administered caerulein to *Ripk3-* or *Mlkl-*deficient mice (Ripk3^−/−^ or Mlkl^−/−^ mice, respectively) and assessed the roles of necroptosis in AP. We found that Ripk3^−/−^ mice had significantly more severe pancreatic edema and inflammation associated with macrophage and neutrophil infiltration than control mice. Consistently, Mlkl^−/−^ mice were more susceptible to caerulein-induced AP, which occurred in a time- and dose-dependent manner, than control mice. Mlkl^−/−^ mice exhibit weight loss, edematous pancreatitis, necrotizing pancreatitis, and acinar cell dedifferentiation in response to tissue damage. Genetic deletion of *Mlkl* resulted in downregulation of the antiapoptotic genes *Bclxl* and *Cflar* in association with increases in the numbers of apoptotic cells, as detected by TUNEL assay. These findings suggest that RIPK3 and MLKL-mediated necroptosis exerts protective effects in AP and caution against the use of necroptosis inhibitors for AP treatment.

## Introduction

Necroptosis is a necrotic form of programmed cell death that is involved in various inflammatory pathologies^1,2^. The protein kinase receptor-interacting protein kinase 1 (RIPK1) is an upstream signaling molecule that is shared by both apoptosis and necroptosis, and caspase 8 is the critical switch that determines whether cells die by apoptosis or necroptosis^2–5^. Upon binding of ligands to cell surface receptors such as Toll-like receptors or tumor necrosis factor receptors, RIPK1 interacts with receptor-interacting protein kinase-3 (RIPK3) through the RIP homotypic interaction motif to form a necrosome complex^5^. Phosphorylated RIPK3 phosphorylates mixed-lineage kinase domain-like (MLKL) through a C-terminal kinase-like domain^6,7^. Phosphorylated MLKL undergoes a conformational change in the N-terminal four-helix bundle domain, which forms a tetrameric complex^2,6^. The MLKL tetramer further translocates to the plasma membrane and oligomerizes to induce pore formation, resulting in sodium influx, osmotic pressure changes, and cell lysis^8^. The release of intracellular proteins, including various damage-associated molecular patterns, creates a microenvironment that recruits inflammatory immune cells, leading to uncontrolled inflammation^6^. Recent studies have demonstrated the contribution of necroptosis to various inflammatory diseases^2,9,10^, and we have revealed that increased necroptosis in type II alveolar epithelial cells causes pulmonary fibrosis^11^. Thus inhibition of necroptosis has been extensively studied as a potential therapeutic strategy for disease-specific applications^12^.

Acute pancreatitis (AP) is a local inflammatory reaction in the pancreas involving cholecystokinin-stimulated pancreatic enzyme secretion that damages pancreatic tissue^13,14^, and elevations in serum levels of amylase and lipase are correlated with the disease^13^. Although severe AP is fatal, a disease-specific treatment has not yet been identified. The severity of the disease is closely associated with massive acini necrosis^13,14^. Regarding the contribution of necroptosis to the pathophysiology of AP, it is unclear whether inhibition of necroptosis mediated by RIPK3/MLKL is effective in protecting against pancreatic damage because different outcomes have been reported. Inhibition of necroptosis with the RIPK1 inhibitor necrostatin-1 or by genetic deletion of either *Ripk3* or *Mlkl* provides protection against AP by reducing acinar cell vacuolization and necrosis^15–19^. In contrast, necrotizing pancreatitis is accelerated in *Ripk3*-deficient mice^20^. Thus, we investigated the roles and consequences of RIPK3 and the MLKL-mediated necroptosis pathway in AP by using *Ripk3-* or *Mlkl*-deficient mice (Ripk3^−/−^ or Mlkl^−/−^ mice, respectively). We induced experimental AP in Ripk3^−/−^ and Mlkl^−/−^ mice via administration of excessive doses of caerulein. Ripk3^−/−^ and Mlkl^−/−^ mice exhibited greater pancreatic edema and recruitment of inflammatory cells into the pancreas than control mice. Inhibition of MLKL-driven cell death resulted in increased apoptosis associated with the expression of antiapoptotic genes. These findings suggest that RIPK3 and MLK-mediated necroptosis play protective roles in AP.

## Materials and methods

### Mice

Female C57BL/6 mice were purchased from Japan SLC (Hamamatsu, Japan). Ripk3^−/−^ and Mlkl^−/−^ mice have been previously described^11^. Six- to 12-week-old mice were used for the experimental and control groups. The mice were housed under specific pathogen-free conditions with unlimited access to food and water in the animal research center of Tokushima University. All animal experiments were performed according to the protocols approved by the animal research committee of Tokushima University and the institution’s guidelines for animal care and use.

### Experimental AP

Mice were randomly divided into control and experimental groups and then fasted overnight. The experimental groups were administered caerulein (Bachem, Bubendorf, Switzerland) by intraperitoneal injection in three different regimens (Supplementary Fig. 1), as follows:

(A) Caerulein (50 µg/kg body weight) was administered every hour for eight consecutive hours, and mice were sacrificed 24 h after the first injection.
(B) Caerulein (50 µg/kg body weight) was administered every hour for six consecutive hours on 2 days separated by 1 day of rest, and mice were sacrificed 24 h after the first injection on the last day.
(C) Caerulein (100 µg/kg body weight) was administered every hour for eight consecutive hours, and mice were sacrificed 24 h after the first injection.

For kinetic experiments, mice were injected with 100 µg/kg body weight caerulein every hour and sacrificed at 2, 4, 8, and 24 h after the first injection. The total mouse body weight was measured before and after treatment. Blood plasma was collected by centrifugation at 3000 r.p.m. for 10 min and was then used for the determination of amylase and lipase activity.

### Serum lipase and amylase

Serum lipase and amylase concentrations were determined by CLEA Japan Inc. (Tokyo, Japan) and Oriental Yeast Co., Ltd (Tokyo, Japan), respectively.

### Histopathological analysis

Pancreatic tissues were fixed in a 10% (v/v) neutral buffered formalin solution (Wako, Osaka, Japan) for 24 h and embedded in paraffin. Paraffin sections were cut and stained with hematoxylin/eosin (H&E). The severity of AP was evaluated by two experienced pathologists blinded to the study protocol. The pancreatic structure, inflammation, and necrosis were semiquantitatively analyzed according to a scoring system described previously (Supplementary Table 1). Apoptotic cells were analyzed using terminal deoxynucleotidyl transferase dUTP nick end labeling (TUNEL) staining according to the manufacturer’s instructions (Abcam, ab206386). For a positive control, sections were treated with 1 µg/µl DNase I in TBS/1 mM MgSO_4_ for 20 min at room temperature. The specimens were examined by light microscopy (Olympus, BX53).

### Isolation of pancreatic cells

Pancreatic tissues were carefully removed and immediately resuspended in 3 ml of Hank’s balanced salt solution (HBSS; Wako, Osaka) on ice. The tissues were cut into small pieces and digested in HBSS containing 2.5 mg/ml collagenase P and 0.1 mg/ml DNase I for 30 min at 37 °C. After incubation, the cells were washed with cold sterile phosphate-buffered saline (PBS) and collected by centrifugation at 1500 r.p.m. for 10 min. The cell pellets were resuspended in a cold HBSS medium, filtered through a 100-µm strainer (BD Falcon, no. 352360), and further collected by centrifugation at 1500 r.p.m. for 10 min. The cells were counted with a hemocytometer, and a Countess II FL automated cell counter (Invitrogen) was used. For edema analysis, whole pancreas samples were weighed and dried at 95 °C for 48 h. Edema was calculated following desiccation and is expressed as a ratio of the wet weight (wet weight  − dry weight/wet weight × 100).

### Flow cytometry

To block the Fc receptor, isolated pancreatic cells were incubated with the 2.4G2 antibody for 15 min at 4 °C and then washed twice in FACS buffer (PBS containing 0.5% FBS, 0.05% NaN_3_). The cells were stained with 50 µl of an antibody cocktail for 15 min at 4 °C in the dark. Live and dead cells were discriminated using 7AAD (BioLegend). Fluorochrome-conjugated antibodies against the following proteins were used: CD3 (BioLegend, 145-2C11), CD4 (BioLegend, GK1.5), CD8 (BioLegend, 53-6.7), CD11b (BioLegend, M1/70), CD11c (eBioscience, N418), CD19 (BioLegend, eBio1D3), F4/80 (BioLegend, BM8), CD45 (BioLegend, 30-F11), Ly6G (BioLegend, 1A8), and CD206 (BioLegend, C068C2). After incubation with antibodies, the stained cells were washed twice in FACS buffer and then fixed with 4% paraformaldehyde (Wako, Osaka, Japan). The data were assessed using the BD FACSCanto II system (BD Biosciences) and analyzed with FlowJo (TreeStar, Ashland, OR).

### Real-time quantitative PCR (qPCR)

Pancreatic tissue was immediately placed in RNAlater^TM^ solution (Invitrogen, no. AM7020) after snap-freezing in liquid nitrogen. Total RNA was first extracted with TRIzol reagent (Takara, no. 9109) followed by a ReliaPrep^TM^ RNA Tissue Miniprep system according to the manufacturer’s instructions (Promega, Madison, WI, USA). RNA integrity was observed using agarose gel electrophoresis. Then, cDNA was synthesized using ReverTra Ace^®^ qPCR RT Master Mix according to the manufacturer’s instructions (Toyobo, Osaka, Japan). mRNA expression was analyzed by qPCR on a Step-One RT-PCR system (Applied Biosystems) using SYBR green incorporation. The raw data were analyzed by the 2^−ΔΔCT^ method and normalized to *Hprt* expression. The primer sequences used in this study are shown in Supplementary Table 2.

### Western blotting

Fresh pancreatic tissues were homogenized in RIPA buffer (Nacalai Tesque, Kyoto, Japan) containing phosphatase inhibitor cocktail (Roche, Basel, Switzerland), protease inhibitor cocktail (Roche, Mannheim, Germany), and 1 mmol/L phenylmethylsulfonyl fluoride (Sigma, St. Louis, USA). Protein concentration was measured using the Pierce BCA Protein Assay Kit (Thermo Scientific, no. 23225). Total protein was denatured in a loading buffer containing SDS at 95 °C for 5 min. Fifty micrograms of denatured protein was loaded and separated on a sodium dodecyl-sulfate polyacrylamide gel electrophoresis gel and transferred to a polyvinylidene difluoride membrane. Membranes were probed with the following antibodies: anti-pRIPK3 (#ab195117, Abcam) and beta-actin (#sc-47778, Santa Cruz). All primary antibodies were used at a dilution of 1:1000. Anti-rabbit IgG horseradish peroxidase and anti-mouse-IgG horseradish peroxidase secondary antibodies were used. All secondary antibodies were used at a dilution of 1:10,000. Detection of the immune complexes was attained by using ECL western blotting detection reagent (Amersham^TM^).

### Statistical analysis

The data are presented as the mean ± standard error of the mean (SEM). Statistical analysis was performed using paired Student’s *t*-tests. A *P* value of <0.05 was considered to indicate significance (*p < 0.05, **p < 0.01, ***p < 0.001).

## Results

### Optimization of the protocol for caerulein hyperstimulation-induced AP

**Fig. 1:**
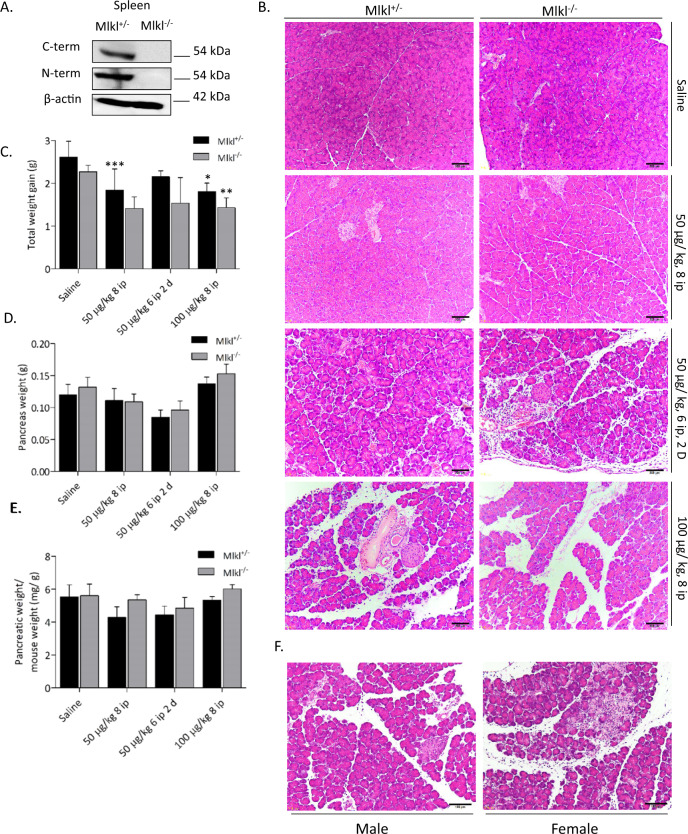
Optimization of the protocol to induce acute pancreatitis in vivo. **A** Western blot of MLKL expression in splenocytes from Mlkl^+/^^−^ and Mlkl^−/−^ mice by using antibodies recognizing the C- and N-terminal domains of MLKL. Mlkl^+/^^−^ and Mlkl^−/−^ mice received intraperitoneal injection of three doses of caerulein, 50 µg/kg/body weight at 8 h intervals, 50 µg/kg/body weight at 6 h intervals for 2 days, and 100 µg/kg/body weight at 8 h intervals. Twenty-four hours after the first injection, mice were sacrificed (*n* = 5−10 in each group). **B** Representative haematoxylin and eosin (H&E) images of pancreases from Mlkl^+/^^−^ and Mlkl^−/−^ mice treated with or without caerulein. **C** Total weight gain (grams), **D** pancreas weight gain (gram), and **E** the ratio of pancreatic weight to the final body weight (milligrams to gram) were determined. **F** Representative H&E images of pancreases from male and female Mlkl^−/−^ mice after receiving caerulein for 8 h. Data are present as means ± SE. *P < 0.05, **P < 0.01, and ***P < 0.001.

We optimized the protocol of caerulein-induced AP and compared the sensitivity of AP between control and Mlkl^−/−^ mice. We first confirmed the absence of MLKL protein in Mlkl^−/−^ mice by using two antibodies recognizing the C- and N-terminal domains of MLKL (Fig. 1A). Next, we optimized the protocol to induce AP in vivo. Mlkl^+/^^−^ and Mlkl^−/−^ mice were starved overnight and then intraperitoneally injected with different doses of caerulein, eight-hourly repeated injections with either 50 or 100 µg/kg of caerulein for one day or eight-hourly repeated injections with 50 µg/kg of caerulein per day for 2 days separated by 1 day of rest. Mice were sacrificed 24 h after the first injection (Supplementary Fig. 1A–C). The untreated group typically had normal pancreatic structure. Histological investigations showed that AP severity increased in a dose-dependent manner (Fig. 1B). Compared to control mice, Mlkl^−/−^ mice receiving caerulein at a dose of 100 µg/kg developed the most severe AP (Fig. 1B). Body weight was significantly decreased in Mlkl^−/−^ mice receiving caerulein at a dose of 100 µg/kg (Fig. 1C). Pancreatic weight and the pancreas-to-body weight ratio tended to be elevated in caerulein-treated Mlkl^−/−^ mice at a dose of 100 µg/kg (Fig. 1D, E). Thus, we administered high-dose cearulein (100 µg/kg of caerulein for 1 day or eight-hourly repeated injections) in the following experiments because this protocol induced the most severe form of AP. Moreover, we evaluated the impact of sex differences on severely injured outcomes to determine the protocol because previous papers have used male and female mice for caerulein-induced AP models^21–23^. We did not see the sex difference in our protocol C (Fig. 1F). Thus, we used female mice in the following experiments.

### MLKL-mediated necroptosis promotes AP

**Fig. 2:**
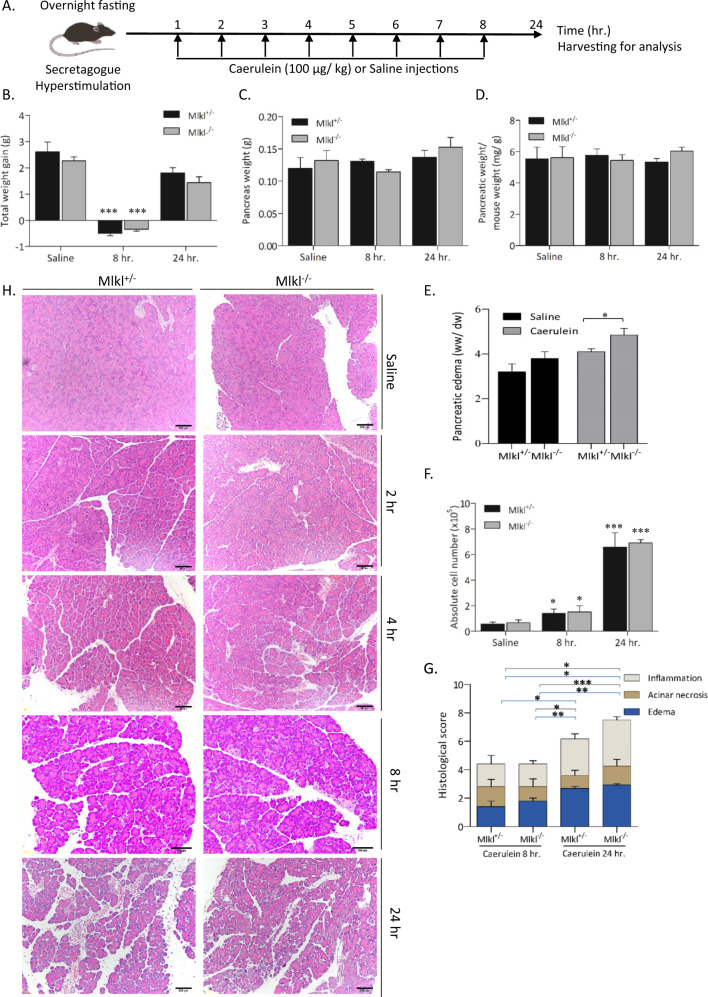
Mlkl^−/−^ mice showed increased inflammation after caerulein treatment. **A** Schematic diagram of caerulein-induced experimental AP in vivo. Mlkl^+/^^−^ and Mlkl^−/−^ mice were intraperitoneally injected with either 100 µg/kg caerulein or saline every hour for eight consecutive hours. The mice were sacrificed at 2, 4, 8, and 24 h after the first injection. The total weight gain (**B**), pancreatic weight (**C**), and the ratio of pancreatic weight to final body weight (**D**) were determined. **E** Pancreatic edema formation was determined by the wet/dry weight ratio in mice sacrificed at 3 h after the last injection. The results are representative of three independent experiments with a minimum of five mice per group. **F** The cell number in each group was determined by an automated cell counter. **G**, **H** Representative H&E images of the pancreas and histological scores of pancreatic edema, necrosis, and inflammation were evaluated in mice sacrificed 8 and 24 h after the first injection; saline or 100 µg/kg caerulein was injected every hour for eight consecutive hours (*n* = 6−15 in each group). The data are presented as the means ± SEMs. *P < 0.05, **P < 0.01, and ***P < 0.001. These data are representative of three experiments.

The pseudokinase MLKL is a key protein that regulates necroptosis^24,25^. MLKL phosphorylated by RIPK3 forms oligomers and translocates to the cell membrane, where it causes pore formation^2,24^. We first investigated whether deletion of *Mlkl* attenuates AP. Mlkl^+/^^−^ and Mlkl^−/−^ mice received repeated injections of 100 µg/kg caerulein every hour for 8 h (Fig. 2A). The pancreatic tissues were harvested 2, 4, 8, and 24 h after the first injection. We observed that mice at 8 h post treatment (early) had a significant body weight loss compared to control mice (Fig. 2B). At 24 h (late) after treatment, Mlkl^−/−^ mice showed a trend towards higher pancreatic weight and a higher pancreas-to-body weight ratio (Fig. 2C, D). A similar result was observed in the wet-to-dry weight ratio, which showed a significant increase in pancreatic edema (Fig. 2E). The absolute cell numbers in the caerulein-treated mice were slightly elevated at 8 h post treatment and significantly peaked at 24 h post treatment (Fig. 2F), but there were no differences among the treatment groups. Histological investigations showed that AP severity increased in a time-dependent manner (Fig. 2G, H). The caerulein-treated mice progressively developed edematous pancreatitis and inflammation from 8 to 24 h after the first injection (Fig. 2G, H). The pathological scores showed a tendency toward more edema, acinar necrosis, and inflammation in Mlkl^−/−^ mice than in control mice, but there was no statistical significance at either the early (8 h) or late (24 h) time point of the specimen collection. These results indicate that Mlkl^−/−^ mice tended to be more susceptible to caerulein-induced AP than control mice.

### *Mlkl* deficiency increased the expression of dedifferentiation-related genes

**Fig. 3:**
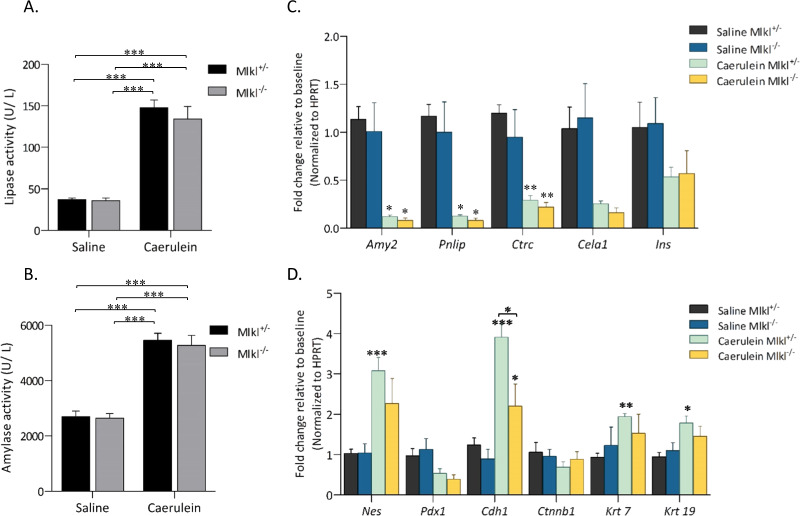
The expression of pancreatic digestive enzymes. Serum lipase (**A**) and amylase activity (**B**) were determined in serum collected after 12 h of treatment with saline or caerulein (*n* = 5−6 in each group). The results of qPCR analysis of mRNA expression in pancreatic tissue are shown in **C**, **D**. Pancreatic digestive enzyme genes (*Amy2*, *Pnlip*, *Ctrc*, *Cela1*, and Ins), genes associated with pancreatic development (*Nes* and *Pdx1*), and ductal and epithelial cell-associated genes (*Cdh1*, *Ctnnb1*, *Krt7*, and *Krt19)* were examined (*n* = 6 in each group). The data are presented as the means ± SEMs. *P < 0.05, **P < 0.01, and ***P < 0.001 compared to the control group. These data are representative of three experiments.

We next investigated the activity of pancreatic lipase and amylase in Mlkl^−/−^ mice. The results showed increased serum levels of lipase and amylase in caerulein-treated Mlkl^−/−^ and control mice, but the differences were not significant (Fig. 3A, B). As Mlkl^−/−^ mice were sensitized to secretagogue-induced pancreatitis, we assessed the expression of genes associated with pancreatic functions and cell-specific markers (Fig. 3C, D). qPCR revealed significantly decreased expression levels of the pancreatic digestive enzymes amylase (*Amy2*), lipase (*Pnlip*), elastase (*Celal)*, chymotrypsinogen (*Ctrc*), and insulin (*Ins*), but there were no differences between caerulein-treated Mlkl^−/−^ and control mice (Fig. 3C). We then analyzed the expression of pancreatic tissue repair markers^26^, including Nestin (*Nes*), Pdx1 (*Pdx1*), E-cadherin (*Cdh1*), and Cyclophilin (*Ctnnb1*). We detected lower E-cadherin expression in Mlkl^−/−^ mice than in control mice (Fig. 3D). The expression of ductal markers, including cytokeratin 7 (*Krt7*) and cytokeratin 19 (*Krt19*), was comparable between Mlkl^−/−^ and control mice (Fig. 3D). These data suggested that the reduced E-cadherin levels in pancreatic tissue in Mlkl^−/−^ mice were involved in severe acinar cell damage and pancreatic inflammation.

### *Mlkl* deficiency reduces neutrophil infiltration associated with AP in mice

**Fig. 4:**
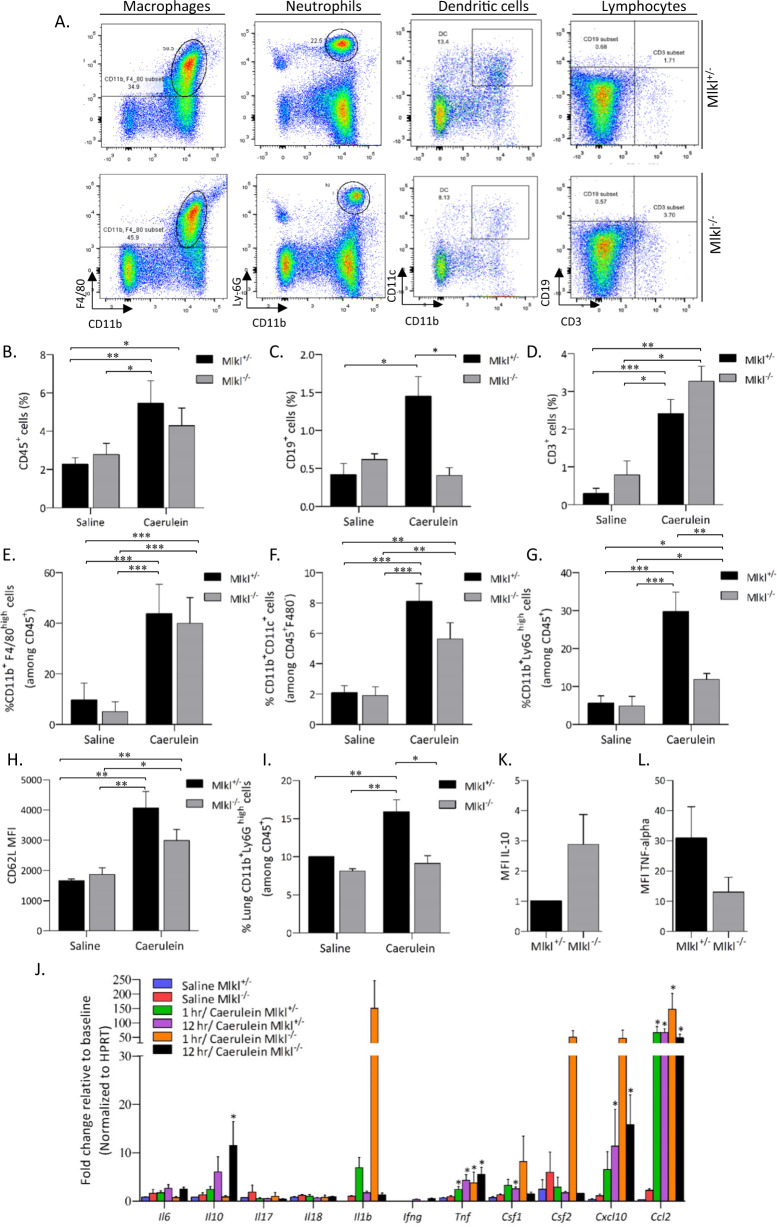
Low neutrophil infiltration was associated with mild AP in Mlkl^−/−^ mice. **A** Representative dot plot of flow cytometry for the frequencies of CD45^+^CD11b^+^F4/80^high^ macrophages, CD45^+^CD11b^+^Ly6G^high^ neutrophils, CD45^+^F4/80^−^CD11b^+^CD11c^+^ dendritic cells, and lymphocytes. Bar plots showing the frequency of CD45^+^ cells (**B**), the frequency of CD3^−^CD19^+^ B cells (**C**), the frequency of CD3^+^ T cells (**D**), the frequency of CD45^+^CD11b^+^F4/80^high^ macrophages (**E**), the frequency of CD45^+^F4/80^−^CD11b^+^CD11c^+^ dendritic cells (**F**), the frequency of pancreatic CD45^+^CD11b^+^Ly6G^high^ neutrophils (**G**), the frequency of lung CD45^+^CD11b^+^Ly6G^high^ neutrophils (**I**), the mean fluorescence intensity (MFI) of CD62L on neutrophils (**H**), of TNF-α and IL-10 on CD45+CD11b+F4/80_high_ macrophages (**J**, **K**). The data are presented as the means ± SEMs (*n* = 10−15 mice/genotype). *P < 0.05, **P < 0.01, and ***P < 0.001. **L** qPCR analysis of proinflammatory cytokine (*Il1b*, *Il6*, *Il17*, *Il18*, *Ifng*, *Tnf*, *Il10*, *Csf1*, and *Csf2*) and chemokine (*Ccl2* and *Cxcl10*) mRNA expression in pancreatic tissue (*n* = 5 in each group) collected at 8 and 24 h after the first injection. *P < 0.05, **P < 0.01, and ***P < 0.001 compared to the control group.

Inflammatory cells in freshly isolated pancreatic tissue were analyzed by flow cytometry (Fig. 4A). The percentage of CD45^+^ cells tended to be higher in caerulein-treated Mlkl^+/^^−^ mice than in Mlkl^−/−^ mice (Fig. 4B). We also detected increased infiltration of dendritic cells, neutrophils, and macrophages at 8 h after the first injection (Supplementary Fig. 2). A small but significant increase in the percentage of CD3^−^CD19^+^ cells was detected in treated Mlkl^+/^^−^ mice compared with Mlkl^−/−^ mice 24 h after the first injection (Fig. 4C), but the frequencies of CD3^+^ cells, macrophages, and dendritic cells were comparable among the treatment groups (Fig. 4D, E and F). The frequencies of neutrophils were significantly higher (more than 2.5-fold higher) in caerulein-treated Mlkl^+/^^−^ mice than in Mlkl^−/−^ mice, but there were no differences in CD62L expression among the treatment groups (Fig. 4G, H). A previous study reported an association of the severity of pancreatitis with lung inflammation. Thus, we evaluated neutrophils in the lungs of caerulein-treated Mlkl^+/^^−^ and Mlkl^−/−^ mice and found decreased numbers of neutrophils in Mlkl^−/−^ mice (Fig. 4I).

We then evaluated inflammatory cytokine expression in macrophages or pancreatic tissues at 8 or 24 h after the first treatment. We detected a tendency towards increased *Il1b*, *Csf*, *Cxcl10*, and *Ccl2* expression expression in the total pancreatic tissue of caerulein-treated Mlkl^−/−^ mice at 8 h after the first injection (Fig. 4J) and detected significantly increased *Il10* and *Tnf* expression at 24 h after the first injection (Fig. 4J). Elevated expression of IL-10 but not TNF-α was detected in macrophages (Fig. 4K, L). These data suggest that mice lacking *Mlkl* developed AP because of increased proinflammatory cytokine- and chemokine-mediated recruitment of dendritic cells and macrophages into the inflamed pancreas.

### Deficiency of *Ripk3* exacerbates AP

**Fig. 5:**
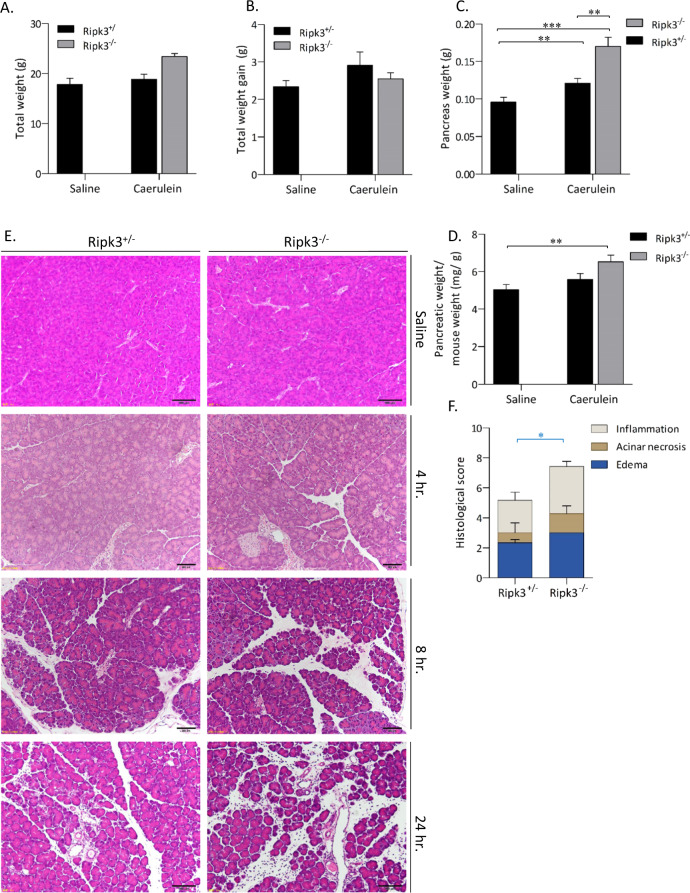
Ripk3^−/−^ mice exhibited exacerbated pancreatic tissue damage in vivo. Ripk3^+/−^ and Ripk3^−/−^ mice were intraperitoneally injected with either 100 µg/kg caerulein or saline every hour for eight consecutive hours. The mice were sacrificed at 4, 8, and 24 h after the first injection (*n* = 6−8 in each group). **A** The total body weight and **B** weight gain were determined. The pancreas weight is shown as the absolute weight (**C**) or as a percentage of the final body weight (**D**). **E** Representative H&E images of pancreas tissue. **F** Histological scores of pancreatic sections from Ripk3^+/−^ and Ripk3^−/−^ mice treated with caerulein showing pancreatic edema, acinar necrosis, and inflammation. There were no significant differences in any parameters such as edema, necroptosis, and inflammation. The data are presented as the means ± SEMs. *P < 0.05, **P < 0.01, and ***P < 0.001. These data are representative of three experiments.

RIPK3 kinase activity is essential for the regulation of MLKL-mediated necroptosis^1,21,22^. Since Mlkl^−/−^ mice developed stronger AP and pancreatic inflammation than control mice, we tested whether the lack of RIPK3 expression affects AP. To examine whether RIPK3-dependent necroptosis contributes to the disease in vivo, Ripk3^−/−^ mice were administered 100 µg/kg caerulein every hour for 8 h. Twenty-four hours after the first administration, the results revealed no difference in body weight gain between saline- and caerulein-treated mice (Fig. 5A, B). AP manifested as a significant increase in absolute pancreatic weight (Fig. 5C) and pancreatic edema (Fig. 5D) in caerulein-treated Ripk3^−/−^ mice compared with control mice. We observed acinar necrosis and pancreatic edema 8 h after the first injection, but there were no differences in caerulein-treated control and Ripk3^−/−^ mice (Fig. 5E). Blinded pathological examination revealed that Ripk3^−/−^ mice exhibited higher histological scores than control mice (Fig. 5E, F). These data indicated that Ripk3^−/−^ mice exhibited edematous pancreatitis and necrotizing pancreatitis, which leads to more severe AP in these mice than in control mice.

### Ripk3^−/−^ exacerbates inflammation associated with experimental AP in mice

**Fig. 6:**
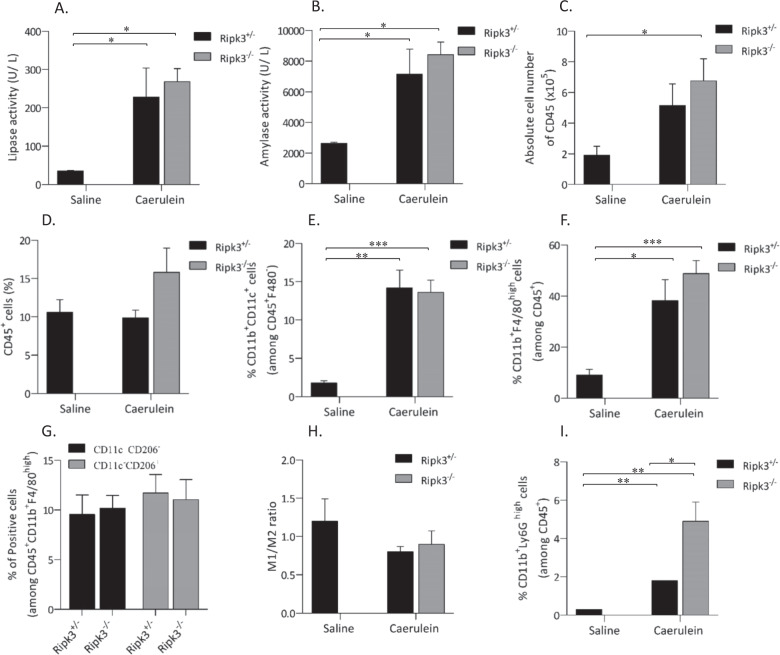
The severity of AP in Ripk3^−/−^ mice was associated with neutrophil infiltration. Lipase (**A**) and amylase activity (**B**) were determined in serum collected at 12 h after the last injection of either PBS or caerulein. **C** Absolute CD45^+^ cell counts in freshly isolated pancreatic tissues from each group were determined by an automated cell counter. Flow cytometry analysis was performed to identify inflammatory immune cells in pancreatic tissue. Bar plots showing the percentage of CD45^+^ cells (**D**), the percentage of CD45^+^F4/80^−^CD11b^+^CD11c^+^ dendritic cells (**E**), the percentage of CD45^+^CD11b^+^F4/80^high^ macrophages (**F**), the percentages of CD11c^+^CD206^-^ M1-like macrophages and CD11c^−^CD206^+^ M2-like macrophages (**G**), the ratio of M1/M2 macrophages (**H**), and the percentage of CD45^+^CD11b^+^Ly6G^high^ neutrophils (**I**). The data are presented as the means ± SEMs. *P < 0.05, **P < 0.01, and ***P < 0.001. These data are representative of three experiments.

We then analyzed amylase and lipase activity and immune cell infiltration into pancreatic tissue in Ripk3^−/−^ mice. Mice treated with caerulein exhibited significant increases in serum amylase and lipase activity and three- and seven-fold increases in serum amylase and lipase levels, respectively (Fig. 6A, B). There were no statistically significant differences in serum pancreatic amylase and lipase levels between caerulein-treated Ripk3^+/−^ and Ripk3^−/−^ mice (Fig. 6A, B). Flow cytometry analysis showed that caerulein-treated control mice had significantly increased CD45^+^ immune cell infiltration in pancreatic tissue (Fig. 6C, D). The percentages of dendritic cells (CD45^+^F4/80^−^CD11b^+^CD11c^+^ cells) and macrophages (CD45^+^F4/80^+^CD11b^+^ cells) were increased by caerulein treatment, but there were no differences between Ripk3^+/^^−^ and Ripk3^−/−^ mice (Fig. 6E, F). The ratio of M1 to M2 macrophages tended to be reduced by caerulein treatment, but there was no difference between Ripk3^−/−^ and control mice (Fig. 6G, H). The elevated levels of pancreatic tissue damage in Ripk3^−/−^ mice were associated with significantly increased neutrophil (CD45^+^CD11b^+^Ly6G^high^) infiltration (Fig. 6I). These data indicated that Ripk3^−/−^ mice exhibited more severe AP with increased edema and more infiltration of neutrophils than control mice, suggesting that Ripk3 has protective roles in AP.

### Mlkl^−/−^ increases apoptosis in acinar cells in mouse pancreatic tissue

**Fig. 7:**
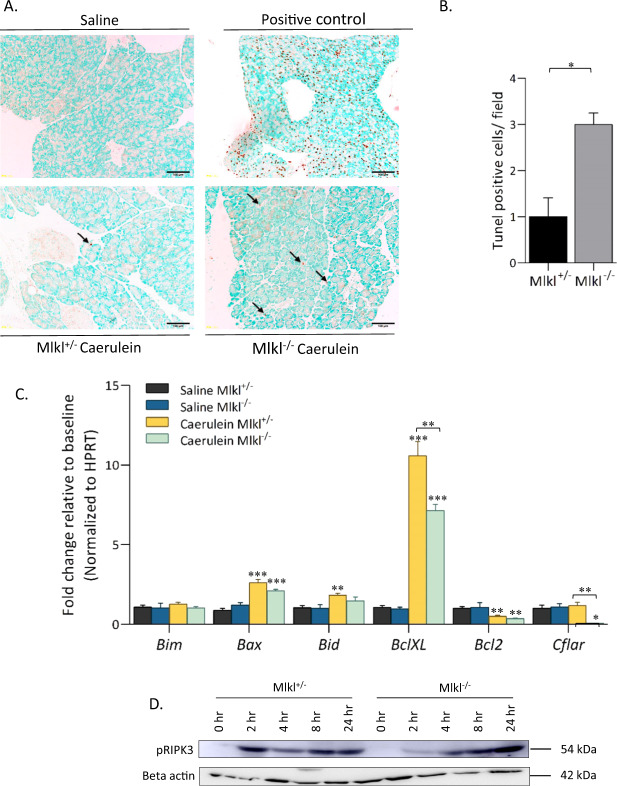
Caerulein-induced AP leads to a higher level of apoptosis in Mlkl^−/−^ mice than in Mlkl^+/^^−^ mice. Pancreatic apoptosis was detected by terminal deoxynucleotidyl transferase dUTP nick end labeling (TUNEL) staining. **A** Representative images of TUNEL-stained pancreatic sections from Mlkl^−/−^ and Mlkl^+/^^−^ mice treated with either saline or caerulein. Pancreatic sections were incubated with DNase I as a positive control (**B**). The number of TUNEL-positive cells per field. **C** qPCR analysis of the mRNA expression of proapoptotic genes (*Bim*, *Bax*, and *Bid*) and antiapoptotic genes (*BclXL*, *Bcl2*, and *Cflar*) in pancreatic tissue after 12 h of treatment with saline or caerulein (*n* = 6 in each group). **D** Kinetics of pRIPK3 during the course of cearulein treatment, mouse pancreatic tissue was collected at the indicated times and then cell lysates (50 μg of protein) were analyzed by immunoblotting. The data are presented as the means ± SEMs. *P < 0.05, **P < 0.01, and ***P < 0.001 compared to the control group.

We assessed whether the loss of necroptosis in Mlkl^−/−^ mice was associated with apoptosis in acinar cells. A TUNEL assay showed a significant increase in the number of apoptotic cells in the pancreas in caerulein-treated Mlkl^−/−^ mice (Fig. 7A, B). We then analyzed apoptosis-related mRNA expression 24 h after the first treatment. The caerulein-treated Mlkl^+/^^−^ mice had increased mRNA expression levels of antiapoptotic genes, including *Bclxl* and *Cflar* (Fig. 7C). We next investigated the kinetics of RIPK3 phosphorylation. The caerulein-treated Mlkl^+/^^−^ and Mlkl^−/−^ mice had high levels of phosphorylated RIPK3 at all time points but there were no significant differences between the two (Fig. 7D). These data suggested that apoptosis occurrence was increased in acinar cells in caerulein-treated Mlkl^−/−^ mice and this effect might have been attributable to the low expression of antiapoptotic genes caused by *Mlkl* deficiency.

## Discussion

AP is characterized by unpredictable inflammation of the pancreas, and in progressive severe cases, systemic complications develop that are associated with high mortality rates^27–30^. Improved treatment strategies for AP that are safer, more effective, and less invasive than existing approaches are urgently needed. Currently, targeted inhibition of necroptosis is being explored as a therapeutic strategy for inflammatory diseases^12^ because various studies have reported the contribution of necroptosis to inflammatory responses^2,9–11^. Thus, we investigated the roles of necroptosis in a mouse AP model in which genes related to necroptosis, specifically *Ripk3* and *Mlkl*, were deleted. Compared with control mice, Ripk3^−/−^ and Mlkl^−/−^ mice exhibited increased pancreatic edema and recruitment of inflammatory cells together with increased apoptosis of acinar cells. These findings suggest that necroptosis exerts protective effects in AP by suppressing apoptosis in acinar cells.

In this study, we investigated caerulein-induced pancreatic tissue damage in either Ripk3^−/−^ or Mlkl^−/−^ mice. We obtained several lines of evidence indicating that the absence of necroptotic regulators sensitizes cells to the development of AP triggered by pancreatic exocrine signaling. First, deletion of *Ripk3* led to increased cell swelling and inflammatory cell infiltration. Second, caerulein-treated Mlkl^−/−^ mice exhibited reduced body weights, increased pancreatic edema, and enhanced mRNA expression of proinflammatory cytokines and chemokines in the early phase of AP. The upregulation of these genes was associated with elevations in the numbers of infiltrating macrophages and dendritic cells. There were slight phenotypic differences between Ripk3^−/−^ and Mlkl^−/−^ mice such as increased neutrophil infiltration in Ripk3^−/−^ mice and decreased neutrophil infiltration in Mlkl^−/−^ mice. RIPK3 regulates not only necroptosis but also apoptosis and other cellular functions including those related to NLRP3 inflammasomes^3,31^, which might explain the phenotypic differences between Ripk3^−/−^ and Mlkl^−/−^ mice. In addition, we observed that caerulein-treated Mlkl^−/−^ mice showed significantly lower levels of Cdh1 expression than control mice. Although we still do not know how necroptosis affects *Cdh1* expression, it might be possible that the lower level of pancreatic tissue repair in Mlkl^−/−^ mice with AP reflects less sensitivity to acinar cell damage and pancreatic inflammation.

Previous studies have reported contradictory results regarding the roles of necroptosis in AP. Several studies have reported that inhibition of RIPK3 and MLKL leads to the protection of AP by reducing acinar cell vacuolization and necrosis^15–19^, while another study has reported that AP development is accelerated in the absence of RIPK3^20^. Here, we evaluated AP by using mice in which *Ripk3* or *Mlkl* was deleted and found that the absence of either gene worsened the AP outcome. One possible explanation for the distinct outcomes would be the different treatment schedules and amounts of caerulein used in these studies. If the schedule or dose of caerulein is the major reason, necroptosis may contribute to AP pathology only in a particular time frame or restricted pancreatic area. If the window for observing the roles of necroptosis is very small, the outcome may fluctuate depending on the dose and timing of caerulein administration.

We detected upregulation of *Tnfα* and *Mcp1* at 1 h after treatment and a high level of *Il10* mRNA expression at 12 h after treatment. These changes were associated with the enhanced migration of macrophages, but not neutrophils into the pancreas in Mlkl^−/−^ mice. One study suggested that IL-10 secreted from M2 macrophages suppresses the migration of neutrophils into the pancreas^32^. As decreased neutrophil migration into the pancreas results in resistance to the development of AP^33^, the elevated expression of *Il10* in macrophages in *Mlkl-*deficient mice might also play roles in the suppression of neutrophil migration.

Blockade of MLKL-driven necroptosis increased apoptosis of acinar cells by TUNEL assay and reduced the expression of antiapoptosis-related genes. RIPK1 is an upstream mediator of the necroptosis cascade, and in the absence of RIPK3, RIPK1 kinase-dependent activity can trigger cell death through FAAD-caspase 8-mediated apoptosis^4^. However, it remains unclear whether the absence of MLKL also shifts the functions of RIPK1 toward increased apoptosis. One possibility is the enhancement of a feedback mechanism to suppress inflammation via increased apoptosis or secondary effects caused by increase in the levels of inflammatory mediators. In addition, as we could not detect cleaved caspase3 and eight in total cell lysates of mice treated with caerulein (data not shown) probably because a few cells undergo apoptosis by TUNEL assay, the roles of apoptosis together with other types of cell death including pyroptosis in the pathophysiology of AP in Mlkl^−/−^ mice require further study in the future.

In conclusion, the present findings suggest that necroptosis plays a protective role in mouse AP. Although conflicting results have been obtained depending on the timing and amount of caerulein, our data caution against the simple use of necroptosis inhibitors for AP treatment. Furthermore, histological analysis of human samples is needed to understand whether the data obtained from mouse studies reflect human AP pathology.

## Supplementary information

Supplementary figures

Supplementary figures legends

Supplementary Table
