# Supplementary figures and images for: Necroptosis protects against exacerbation of acute pancreatitis
Source: Cell Death Dis. 2021 Jun 10;12(6):601. doi: 10.1038/s41419-021-03847-w (PMC8192754; doi:10.1038/s41419-021-03847-w)

Supplementary Figure 1

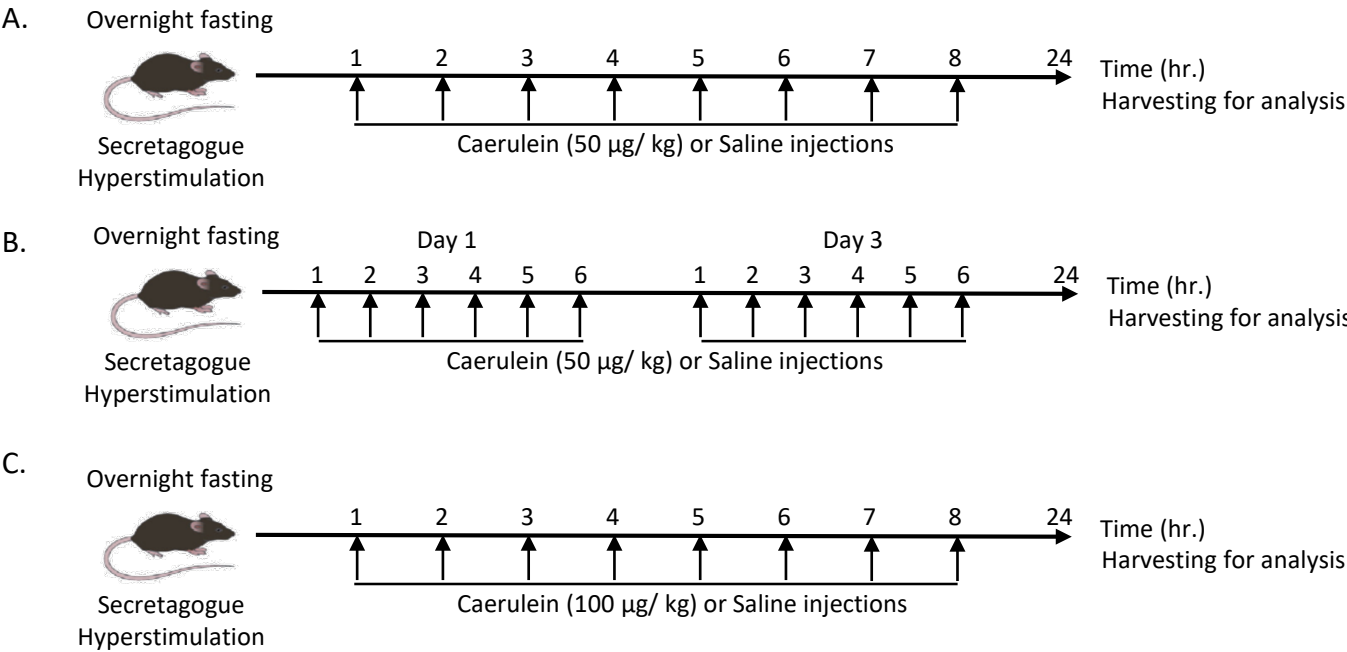

Supplementary Figure 2

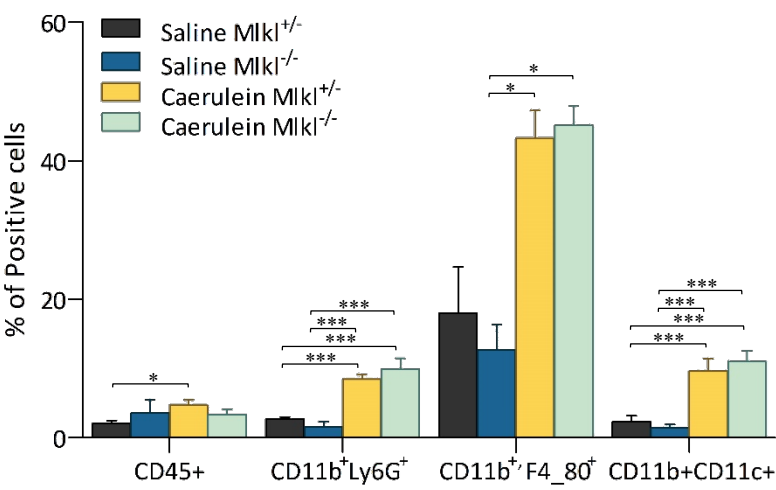

Supplement: Supplementary file 1 — Supplementary figures [file 41419_2021_3847_MOESM1_ESM.pdf]
