# Supplementary material for: Necroptosis protects against exacerbation of acute pancreatitis
Source: Cell Death Dis. 2021 Jun 10;12(6):601. doi: 10.1038/s41419-021-03847-w (PMC8192754; doi:10.1038/s41419-021-03847-w)
Supplement: Supplementary file 2 — Supplementary figures legends [file 41419_2021_3847_MOESM2_ESM.pdf]

Supplementary Figures legends

Supplementary Figure 1. A schematic diagram of the caerulein-induced experimental acute pancreatitis in vivo in this study. Mice were received intraperitoneal injection at three different doses.

(A) Caerulein (50 µg/kg body weight) was administered every hour for eight consecutive hours, and mice were sacrificed 24 hours after the first injection.
(B) Caerulein (50 µg/kg body weight) was administered every hour for six consecutive hours on two days separated by 1 day of rest, and mice were sacrificed 24 hours after the first injection on the last day.
(C) Caerulein (100 µg/kg body weight) was administered every hour for eight consecutive hours, and mice were sacrificed 24 hours after the first injection.

Supplementary Figure 2. $Mlkl^{+/-}$ and $Mlkl^{-/-}$ mice were received intraperitoneal injection at 100 µg/ kg/ bodyweight at seven-hour intervals. One-hour post treatment, mice were sacrificed. Flow cytometry analysis of inflammatory immune cell in pancreatic tissue showing the percentages of $CD45^{+}$ cells, $CD45^{+}F4/80^{-}CD11b^{+}CD11c^{+}$ dendritic cells, $CD45^{+}CD11b^{+}F4/80^{high}$ macrophages, and $CD45^{+}CD11b^{+}Ly6G^{high}$ neutrophils. Data are present as means ± SE (n=10-15 micei /genotypes). * P<0.05, ** P<0.01, and ***P<0.001.
