# Supplementary material for: Necroptosis protects against exacerbation of acute pancreatitis
Source: Cell Death Dis. 2021 Jun 10;12(6):601. doi: 10.1038/s41419-021-03847-w (PMC8192754; doi:10.1038/s41419-021-03847-w)
Supplement: Supplementary file 3 — Supplementary Table [file 41419_2021_3847_MOESM3_ESM.pdf]

Supplementary Table 1. Histological scoring criteria

Parameter	Score	Indication
Edema	0	Absent
	1	Diffuse expansion of interlobar septa
	2	Same as 1 + diffuse expansion of interlobular septa
	3	Same as 2 + diffuse expansion of interacinar septa
	4	Same as 3 + diffuse expansion of intercellular septa
Acinar necrosis	0	Absent
	1	Diffuse occurrence of 1-4 necrotic cells/HPF
	2	Diffuse occurrence of 5-10 necrotic cells/HPF
	3	Diffuse occurrence of 11-16 necrotic cells/HPF
	4	>16 necrotic cells/HPF (extensive confluent necrosis)
Hemorrhage/fat necrosis	0	Absent
	1	2 foci
	2	4 foci
	3	6 foci
	4	≥ 8 foci
Inflammation/perivascular infiltration	0	0-5 interlobular or perivascular leukocytes/HPF
	1	6-10 interlobular or perivascular leukocytes/HPF
	2	11-20 interlobular or perivascular leukocytes/HPF
	3	21-30 interlobular or perivascular leukocytes/HPF
	4	>31 interlobular or perivascular leukocytes/HPF/ microabscesses

HPF, high-power field. The maximum damage score is 16 points.

Supplementary Table 2. Forward and reverse quantitative reverse transcription polymerase chain reaction primers

Gene	Forward	Reverse
Hprt	5'-AGCCTAAGATGAGCGCAAGT-3'	5'-TTACTAGGCAGATGGCCACA-3'
Amy2	5'-CCTTCTGACAGAGCCCTTGTG-3'	5'-GGATGATCCTCCAGCACCAT-3'
Pnlip	5'-AGAGGACCTTTGGAGCCATTGGAA-3'	5'-ATTGCGTCCACAAACTGAGCATCG-3'
Ctrc	5'-AGTTCAACTCCTTCACCGTGCGTA-3'	5'-AGCAGGGAAGTCATCGTCAACAGT-3'
Cela1	5'- CTGAAGCCCGGAGGAACTC-3'	5'-TGGTGCCATGATCCTCCATA-3'
Ins	5'-ACCTGGAGACCTTAATGGGCCAAA-3'	5'-ATGACCTGCTTGCTGATGGTCTCT-3'
Nes	5'-AGCCATTGTGGTCTACGGAAGTGA-3'	5'-AGCACCTCTTGGTTCTCATCCACA-3'
Pdx1	5'-ACTTAACCTAGGCGTCGCACAAGA-3'	5'-TTGGCATCAGAAGCAGCCTCAAAG-3'
Cdh1	5'-TCAAGCTCGCGGATAACCAGAACA-3'	5'-ATTCCCGCCTTCATGCAGTTGTTG-3'
Ctnnb1	5'- TGCAGCTTCTGGGTTCCGATGATA-3'	5'-AGATGGCAGGCTCAGTGATGTCTT-3'
Krt7	5'-TGCAGTCGCAGATCTCAGACACAT-3'	5'- TGTGGTTGGCCATCTCCTCATACT-3'
Krt19	5'-AGTTTGAGACAGAACACGCCTTGC-3'	5'- TCAGGCTCTCAATCTGCATCTCCA-3'
Bim	5'-CACTGGTTGCTGGCTTTGCTG-3'	5'-TCCTTGCTCCTGGAAATGACCTGG-3'
Bax	5'-GGGTGGTTGCCCTCTTCTACTTTG-3'	5'-AGTCCAGTGTCCAGCCCATGATG-3'
Bid	5'-AATCATCCACAACATTGCCAGA-3'	5'-GCCTTGTCGTTCTCCATGCT-3'
BclXL	5'-AACATCCCAGCTTCACATAACCCC-3'	5'-GCGACCCCAGTTTACTCCATCC-3'
Bcl2	5'-GTCCCGCCTCTTCACCTTTCAG-3'	5'-GATTCTGGTGTTTCCCCGTTGG-3'
Cflar	5'-TCCAGAATGGGCGAAGTAAAGAGC-3'	5'-AGTCTCTTCACGATGTGCGGAG-3'
Il6	5'-CACCGGGAACGAAAGAGAAG-3'	5'-CCCAGGGAGAAGGCAACTG-3'
Il10	5'-TGTGAAAATAAGAGCAAGGCAGTG-3'	5'-CATTCATGGCCTTGTAGACACC-3'
Il17	5'-GCTCCAGAAGGCCCTCAGA-3'	5'-CTTTCCCTCCGCATTGACA-3'
Il18	5'-AAAGTTAGGTGGGGAGGGTT-3'	5'-ATGGAAATACAGGCGAGGTC-3'

Il1b	5'-CAACCAACAAGTGATATTCTCCATG-3'	5'-GATCCACACTCTCCAGCTGCA-3'
Ifng	5'-AGCTCATCCGAGTGGTCCAC-3'	5'-AAAATTCAAATAGTGGTGGCAGAA-3'
Tnf	5'-CATCTTCTCAAAATTCGAG-3'	5'-TGGGAGTAGACAAGGTACAACCC-3'
Csf1	5'-ATGGGAGACCAGGACAGATG-3'	5'-TATGCCTTTACGGGAAGTCG-3'
Csf2	5'-GGCCTTGGAAGCATGTAGAGG-3'	5'-GGAGAACTGGTTAGAGACGACTT-3'
Cxcl10	5'-TCCCTATGGCCCTCATTCTCA-3'	5'-CCAAGTGCTGCCGTCATTTC-3'
Ccl2	5'-TTAAAAACCTGGATCGGAACCAA-3'	5'-GCATTAGCTTCAGATTTACGGGT-3'
Hprt	5'-CCAAAATGGTTAAGGTTGC-3'	5'-CCAGTTTCACTAATGACACAAC-3'
